# Hypoxia inducible factor-1 alpha expression is increased in infected positive HPV16 DNA oral squamous cell carcinoma and positively associated with HPV16 E7 oncoprotein

**DOI:** 10.1186/1750-9378-6-18

**Published:** 2011-10-27

**Authors:** Vito Rodolico, Walter Arancio, Marco C Amato, Francesco Aragona, Francesco Cappello, Olga Di Fede, Giuseppe Pannone, Giuseppina Campisi

**Affiliations:** 1 Department of Sciences for Health Promotion, Section of Anatomic Pathology, University of Palermo, Palermo, Italy; 2 Department of Biomedical Internal and Specialized Medicine, Section of Endocrinology, University of Palermo, Palermo, Italy; 3 Department of Experimental Medicine and Clinical Neuroscience, University of Palermo, Palermo, Italy; 4 Department of Surgical and Oncologic Disciplines, Section of Oral Medicine, University of Palermo, Palermo, Italy; 5 Department of Surgical Sciences, Section of Anatomic Pathology and Cytopathology, University of Foggia, Foggia, Italy

**Keywords:** Oral Squamous Cell Carcinoma, Hif-1α, HPV, HPV16, E7

## Abstract

**Background:**

There is increasing evidence for the role of High Risk (HR) Human PapillomaVirus (HPV) in the pathogenesis of Oral Squamous Cell Carcinoma (OSCC). The E6 and E7 oncogenes from HR HPVs are responsible for the deregulation of p53 and pRB proteins involved in cell cycle and apoptotic pathways. In cell lines experiments, the HPV E7 protein seems to be able to enhance Hypoxia Inducible Factor-1 alpha (HIF-1α) activity, normally involved in the response to hypoxia and able to enhance angiogenesis.

**Results:**

We studied tumor specimens from 62 OSCC; a higher prevalence of tumors in TNM stage II and also in pT2 class between OSCC infected positive HPV16 DNA than non-infected ones was observed. HIF-1α positivity was detected throughout the analysed fields, not associated with areas of necrosis and also observed in cells immediately adjacent to blood vessels. A significant increase in mean values of the HIF-1α labeling indexes was observed for pT1-T2, as well for stage I-II, in the infected positive HPV16 DNA tumors than non-infected ones. HIF-1α and HPV16 E7 labeling indexes showed a significantly positive correlation which suggested a positive association between HPV16 E7 and HIF-1α expression.

**Conclusions:**

In our specimens HIF-1α immunoreactivity hints for an O_2_-independent regulatory mechanism in infected positive HPV16 DNA tumors, especially for pT1-T2 and stage I-II tumors, suggesting a very early involvement in the development of HPV-induced OSCC. HIF-1α and HPV16 E7 labeling indexes suggest also a positive association between the two proteins in infected positive HPV16 DNA OSCC.

## Background

Nowadays there is increasing evidence for the role of high risk (HR) Human PapillomaVirus (HPV) in the pathogenesis of Oral Squamous Cell Carcinoma (OSCC) [1-7], till to consider HPV positive (HPV+) OSCC as a distinct clinicopathological entity with a different outcome in comparison with HPV negative (HPV-) OSCC [2,6,8-11]. The HR types HPV16, 18, 31 and others, are frequently found in cervical cancers and have been found to be risk factors also for OSCC, independently from alcohol and tobacco use [2,12-16]. HPV16, the most common HR HPV type detected in biopsies from women with cervical squamous cell carcinoma (55%) [17,18], was also the most common type detected in OSCC (16%), accounted for 68.2% of all HPV+ OSCC [19,20].

The E6 and E7 oncogenes from HR HPVs are responsible for maintaining differentiating cells active in cell cycling and are able to transform both cervical and upper aero-digestive tract epithelia via expression of the viral oncoproteins E6 and E7 and the following deregulation of cell cycle and apoptotic pathways [21,22]. These 2 proteins promote the degradation of cellular tumor suppressors: p53 in the case of E6 and pRb family members in the case of E7. Binding and degradation of pRb family members by E7 results in the release of E2F transcription factors that in turn drive the cell into S phase. The abrogation of pRb function by HR E7 proteins induces a stress response leading to an elevated activity of p53, which can in turn induce apoptosis [23]. But the high-risk E6 proteins degrade p53, thus preventing apoptosis and allowing continued proliferation. In addition to these well-known activities, both proteins have a wide range of other targets [23], and the extent to which these additional interactions contribute to HPV-associated carcinogenesis is not fully understood. Among the additional factors bound by E7 are histone deacetylases (HDAC), which catalyze the deacetylation of histones and other transcriptional regulatory proteins [24].

One important characteristic of both benign and malignant lesions is the ability to promote angiogenesis, which allows a growing lesion to access nutrients and oxygen for growth [25]. Angiogenesis is mainly triggered by hypoxia or reduced tissue oxygen levels. The cellular response to hypoxia is primarily regulated through the activity of the transcription factor Hypoxia-Inducible Factor-1 (HIF-1) [26,27]. HIF-1 consists of a heterodimer of two proteins (HIF-1α and HIF-1β) which mediates the transcription activation of several genes involved in stress response [28]; under normal oxygen conditions (normoxia), the HIF-1α subunit has a very short half-life due to oxygen-dependent hydroxylation and consequent degradation through the von Hippel-Lindau (VHL)/proteasome pathway; under hypoxic conditions, reduced oxygen levels result in the accumulation of HIF-1α, which translocates to the nucleus and leads to the accumulation of functional HIF-1 Transcription Factors that in turn activate the expression of HIF-1 target genes, including a range of proangiogenic factors and enzymes that favor glycolytic over aerobic metabolism [27]. In addition to this hypoxia-dependent stabilization system, a range of other post translational modifications and signaling pathways, also affect HIF-1α synthesis, stability, and activity such as the p53, p300/CBP, and several HDACs [29-31].

Recent studies indicate that, in cell lines experiments, the HPV E7 protein is responsible for enhanced HIF-1α activity and enhances HIF-1 dependent transcription by inducing the dissociation of HDACs from HIF-1α [32].

The aim of this study was to examine the HIF-1α protein expression in OSCC to test the hypothesis that HPV E7 protein may influence HIF-1α expression in OSCC infected positive for HPV16 DNA.

## Results

### Characteristics of the Study Population

We studied tumor specimens from 62 OSCC; clinico-pathological characteristics for non-infected (Group I) vs. infected positive HPV16 DNA tumors (Group II) are detailed in Table 1. The two groups of tumors did not differ statistically significantly with regard to patient age at diagnosis, sex, site, histological grading; instead, statistically significant differences were found regarding TNM stage (*p *< .001) and tumor size (*p *< .001): in particular a higher prevalence of tumors in TNM stage II between OSCC infected positive HPV16 DNA (76.9%) than non-infected ones (23.1%) was observed and refining the components of TNM staging, also in pT2 class (73.3% infected positive HPV16 DNA tumors).

**Table 1:** Comparison of clinico-pathological parameters in HPV- DNA (group I) and HPV16+ DNA (group II) oral squamous cell carcinomas

Parameters	Group I (HPV-) (%)	n.39	Group II (HPV16+) (%)	n.23	*p* value*
Age at diagnosis, year					
< 53	(10)	4	(4)	1	
53-73	(54)	21	(70)	16	
> 73	(36)	14	(26)	6	*N.S*
Sex					
Female	(46)	18	(43)	10	
Male	(54)	21	(57)	13	*N.S*.
Site of primary tumor					
Tongue	(46)	18	(48)	11	
Buccal Mucosa	(26)	10	(22)	5	
Alveolar Ridge Mucosa	(18)	7	(17)	4	
Palate	(10)	4	(13)	3	*N.S*.
Tumor Histological Grade					
Well (G1)	(57)	22	(65)	15	
Moderate (G2)	(28)	11	(26)	6	
Poor (G3)	(15)	6	(9)	2	*N.S*.
TNM Stage					
I	(64)	25	(22)	5	
II	(8)	3	(44)	10	
III	(13)	5	(30)	7	
IV	(15)	6	(4)	1	< .001
pT					
1	(64)	25	(22)	5	
2	(10)	4	(48)	11	
3	(10)	4	(26)	6	
4	(16)	6	(4)	1	< .001

* Chi-square test and Fisher's exact test; statistical results were considered significant for *p *values < .05 (*N.S. *indicates not significant)

### Immunohistochemical analysis of HPV16 E7 oncoprotein

**Figure 1:**
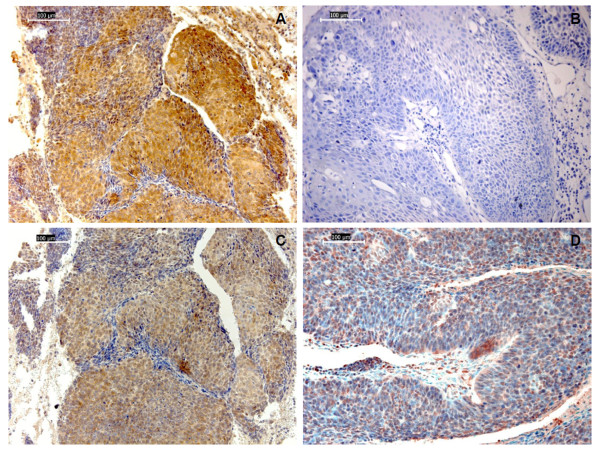
**Representative images of HPV16 E7 oncoprotein and HIF-1α protein immunostaining of paraffin sections from oral squamous cell carcinomas**. A) Tumor positive for HPV16 DNA with almost all epithelial tumor cells stained positive for E7; B) tumor negative for HPV DNA with no HPV16 E7 oncoprotein staining; C) Consecutive section of the same specimen of tumor positive for HPV16 DNA represented in "A)", stained with the anti-HIF-1α antibody: uniform location of immunoreactivity for HIF-1α and HPV16 E7 on the same tumor; D) weak expression for HIF-1α in tumor cells of tumor negative for HPV DNA (magnifications × 200; scale bars 100 μm).

**Figure 2:**
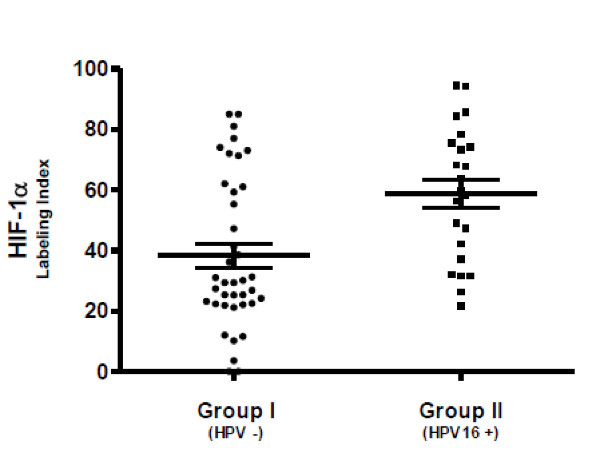
**Labeling indexes immunoreactivity for HIF-1α in paraffin sections from oral squamous cell carcinomas**. A lower HIF-1α protein expression was observed in tumors negative for HPV DNA in comparison with those positive for HPV16 DNA (37.24 ± 25.95 vs. 58.85 ± 22.17; *p *< .001). Differences were analyzed by the Mann-Whitney test; statistical results were considered significant for *p *values < .05.

It was analyzed whether high-risk HPV16 E7 oncoprotein were detectable by immunohistochemical analysis. No HPV16 E7 oncoprotein staining could be detected in the 39 specimens from tumors negative for HPV DNA as expected; instead in the 23 tumors positive for HPV16 DNA, the affinity-purified monoclonal anti-HPV16 E7 antibody recognized almost all epithelial cells within the tumor islets (Figure 1A, B) but did not stain any cells in the adjacent connective tissues. Dysplastic tumor cells in precancerous squamous epithelium adjacent to the cancerous tumor islets in the same section were also positively stained. Keratinocytes in areas of non-neoplastic squamous epithelium were not stained. In keeping with a previous study [33], these findings suggest that E7 can be considered as a marker for premalignant intraepithelial lesions. Whereas all HPV16 DNA positive carcinomas stained positive for E7, a more detailed analysis revealed strong variations in E7 oncoprotein expression levels with labeling indexes ranged from 32.6% to 100% (mean 77.15 ± 25.01). Because there was only one case in pT4 class and in stage IV, we chose to dichotomize size as pT3 and pT4 vs. pT1 and pT2 as well stage TNM III and IV vs. I and II. In the pT1-T2 group, E7 oncoprotein labeling indexes ranged from 32.6% to 95.4% (mean 68.73 ± 19.89); in the pT3-T4 group, E7 oncoprotein labeling indexes ranged from 83,8% to 100% (mean 96.4 ± 6.01); these differences proved to be significant (*p *< .001) (Figure 2). Similar results were also obtained for stages groups [Stage I-II labeling indexes from 32.6% to 96.6% (mean 66.96 ± 19.23); Stage III-IV from 83.8% to 100% (mean 96.27 ± 5.57); *p *< .001]. No significant differences were found for age, sex, site or histological grading (data not shown).

### HIF-1α expression is increased in infected positive HPV16 DNA tumors

Immunohistochemical staining was also performed to identify the presence of the HIF-1α protein; immunoreaction was revealed in all cases but two of the non-infected group, both of them were very small tumors (pT1 of mm 4 and mm 5, TNM stage I) of the tongue.

In our specimens HIF-1α positivity was detected throughout the analysed fields, not associated with areas of necrosis and also observed in cells immediately adjacent to blood vessels. Adjacent non-neoplastic tissue, the stromal and normal cells did not show immunoreactivity for HIF-1α. In the 62 tumor's specimens investigated, the labeling indexes ranged from 0% to 94.5% (mean 45.26 ± 26.6). No significant differences for mean value of HIF-1α labeling indexes were found for age, sex, site or histological grading; however the labeling indexes showed a significant increase in mean values for pT1-T2 tumors vs. pT3-T4 (33.02 ± 19.66 vs. 77.65 ± 9.93; *p *= .015), as well for Stage I-II vs. Stage III-IV (31.41 ± 18.53 vs. 76.6 ± 10.12; *p *= .03).

Generally, immunoreactivity for HIF-1α was detected with intense cytoplasmatic and nuclear positivity in tumor cells of cases with positive HPV16 DNA (Group II) (Figure 1C), whereas a weak cytoplasmatic expression was found in tumor cells of the non-infected group (Group I) (Figure 1D), similar to the one found in some of the islet of precancerous squamous epithelium adjacent to the infected positive HPV16 DNA tumors (Group II).

In the positive HPV16 DNA tumors (Group II), the HIF-1α labeling indexes ranged from 21.7% to 94.5% (mean 58.85 ± 22.17), while in the non-infected group (Group I), ranged from 0% to 85% (mean 37.24 ± 25.95); these differences proved to be significant (*p *< .001) (Figure 2).

**Figure 3:**
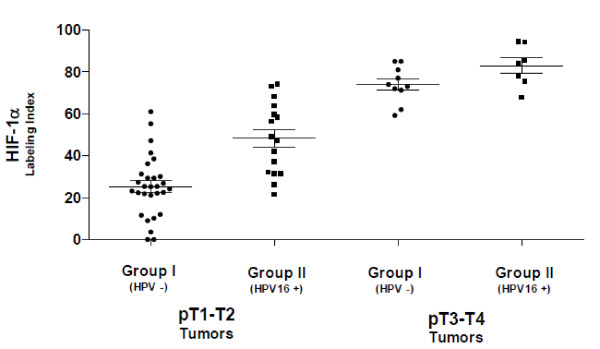
**Labeling indexes immunoreactivity for HIF-1α in pT1-T2 and pT3-T4 oral squamous cell carcinomas**. pT1-T2 tumors showed a significant increase in mean values of the HIF-1α labeling indexes in infected positive HPV16 DNA tumors than non-infected tumors (48.31 ± 17.14 vs. 24.59 ± 15.58; *p *< .001); pT3-T4 tumors did not showed significant differences in mean values of the HIF-1α labeling indexes between infected positive HPV16 DNA tumors and non-infected tumors (82.94 ± 9.77 vs. 73.96 ± 8.64; *p *= .07). Differences were analyzed by the Mann-Whitney test; statistical results were considered significant for *p *values < .05.

On examining the mean values of the HIF-1α labeling indexes between Group II and Group I, significant differences were not found for age, sex, site or histological grading; however, statistical analysis demonstrated for pT1-T2 tumors a significant increase in mean values of the HIF-1α labeling indexes in the infected positive HPV16 DNA tumors (48.31 ± 17.14) than non-infected tumors [(24.59 ± 15.58); *p *< .001], as well for stage I-II tumors [(46.59 ± 16.24 mean value of the HIF-1α labeling indexes in Group II; 23.28 ± 14.17 mean values in Group I); *p *< .001]. The same analysis for pT3-T4 group of tumors [(82.94 ± 9.77 mean values of the HIF-1α labeling indexes in Group II; 73.96 ± 8.64 mean values in Group I); *p *= .07] as well for stage III-IV [(81.85 ± 9.56 mean values of the HIF-1α labeling indexes in Group II; 72.78 ± 9.08 mean values in Group I); *p *= .05] did not showed significant differences in mean values of the HIF-1α labeling indexes between Group II and Group I (Figure 3).

**Figure 4:**
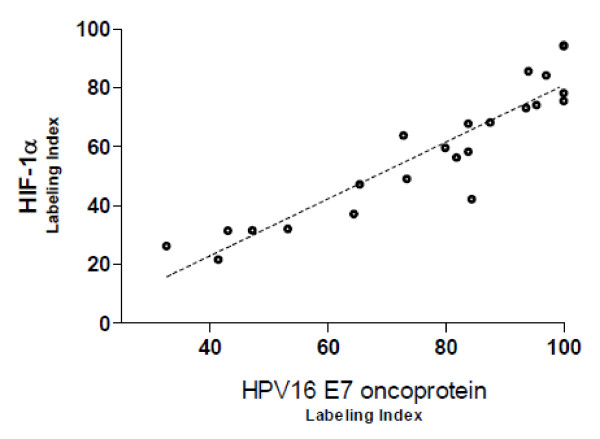
**Bivariate scatter plot of HPV16 E7 and HIF-1α labeling indexes in infected positive HPV16 DNA tumors**. Significantly positive correlation between HPV16 E7 and HIF-1α expression (Rho = .512; *p *< .001. Correlations among continuous variables and *p *values were determined by using the Spearman test; statistical results were considered significant for *p *values < .05.

Interestingly, HIF-1α and HPV16 E7 immunoreactivity were uniform in location on the same tumor (Figure 1A and 1C), and their labeling indexes showed a significantly positive correlation (Rho = .512; *p *< .001), which suggested a positive association between HPV16 E7 and HIF-1α expression (Figure 4). A more detailed analysis showed a stronger positive association between the two proteins in pT1-T2 group than in pT3-T4 [(pT1-T2 tumors: Rho = .669; *p *< .001) (pT3-T4: Rho = .494; *p *= .04)] and stage I-II tumors than stage III-IV [(stage I-II tumors: Rho = .684; *p *< .001) (III-IV: Rho = .510; *p *= .02)].

## Discussion

Several studies have investigated the role of HPV in oral carcinogenesis, confirming that HPV plays a role in oral carcinogenesis and HPV cancers are specific type of tumors with numerous important differences reported in typology of risk patient (generally, never married younger males, < 40 yrs.), histological grading (well differentiated cancer), histotype (possible but not necessary basaloid appearance with a characteristic faster growing) and clinical outcome in term of overall survival (patients with HPV-positive OSCC had a lower risk of dying and a lower risk of recurrence than HPV-negative OSCC patients) [34-37]. However, the protective mechanism by which HPV infection improves the overall survival and prognosis in oral cancer is not yet clearly understood. HPV16, the most common HR HPV type detected in biopsies from women with cervical SCC (55%) [17,18], was also the most common type detected in OSCC (16%), accounted for 68.2% of all HPV+ OSCC [19,20].

In our own study, a higher prevalence of OSCC in TNM stage II was found among infected positive HPV16 DNA tumors than non-infected tumor ones and, refining better the components of TNM staging, a higher prevalence was also found for HPV status in OSCC pT2. The prevalence of OSCC in stage II and pT2 class positively infected by HPV16 could be reasonably explained by their faster growing and higher proliferative activity than negative HPV ones, as also observed in studies investigating markers of proliferation, such as the proliferating cell nuclear antigen (PCNA) [38].

The HR HPV types that infect the genital mucosa typically last from 12-18 months and are eventually cleared by the immune system [39]. Approximately 10% of women fail to clear HPV infections, resulting in long-term persistent infection [40]. Maintenance of the viral episome in basal cells is the basic function of the early, or maintenance, phase of the viral life cycle. E6, E7, E1 and E2 are each necessary for continued episomal maintenance of whole genomes in cell culture [41]. HPVs exert their oncogenic role after DNA integration, gene expression of E5, E6 and E7 loci and p53/pRb host proteins suppression, leading to increased cell proliferation and contributing to carcinogenesis [42,43].

In the present study, the detection of the E7 oncoprotein always coincided with the presence of an invasive OSCC positive for HPV16 DNA; when we compared the E7 oncoprotein expression with the clinico-pathologic characteristics of these lesions, we found an association between E7 oncoprotein expression and TNM stage and tumor size. There is strong evidence that expression of the E7 oncogene is necessary for the induction and maintenance of the transformed phenotype [44]; however, the role of the E7 oncoprotein in OSCC has remained elusive. In this study, we analyzed for the first time the high-risk E7 oncoprotein expression in OSCC infected positive HPV16 DNA and we report the presence of high-risk HPV oncoprotein in all of these OSCC cancer specimens. These findings indicate that the high-risk E7 oncoproteins of HPV16 is expressed continuously in invasive HPV16 DNA positive OSCC.

As HPVs lesions persist they become larger and exhibit increased requirements for nutrients. In proliferating lesions there is competition between cells for nutrients and oxygen, and this can result in arrest of cellular proliferation or even cell death. HPV lesions overcome this constraint by promoting angiogenesis [45-47]. In many cancers, angiogenesis occurs only late in tumor progression, but increased vascular density and production of angiogenic factors is a very early event in the development of HPV-induced pre-malignant lesions and cervical cancers [46,47]. Furthermore, several studies have reported that HPV gene products can induce the production of angiogenic factors from infected cells [48-51].

Transient transfection of E6 and E7 expression vectors into cervical cancer cell lines was reported to induce higher HIF-1α levels under normoxic conditions [48]. Others studies indicate that cells containing complete HPV31 genomes showed enhanced levels of HIF-1α upon treatment with the hypoxia mimic deferoxamine mesylate (DFO), which resulted from protein stabilization and lead to increases in some but not all downstream effectors of the hypoxic response, suggesting that HPV specifically manipulates aspects of the cellular hypoxic response; both HPV E6 and E7 were able independently to enhance induction of HIF-1α upon DFO treatment [52]. A recent study indicate that, in cell lines experiments, the HPV E7 protein is responsible for enhanced HIF-1α activity and enhances HIF-1 dependent transcription by inducing the dissociation of HDACs from HIF-1α [24].

In our specimens HIF-1α positivity was detected throughout the analysed fields, not associated with areas of necrosis and also observed in cells immediately adjacent to blood vessels, suggesting an O_2_-independent regulatory mechanism. Generally, in infected positive HPV16 DNA tumors we found increased expression for HIF-1α, especially for stage I-II and pT1-pT2 tumors, suggesting a very early event in the development of HPV-induced OSCC. We also observed that HIF-1α and HPV16 E7 immunoreactivity were uniform on the same specimens with a positive correlation of their labeling indexes which suggests a positive association between HPV16 E7 and HIF-1α expression in infected positive HPV16 DNA OSCC.

## Conclusions

In our specimens HIF-1α immunoreactivity hints for an O_2_-independent regulatory mechanism in infected positive HPV16 DNA tumors, especially for pT1-T2 and stage I-II tumors, suggesting a very early involvement in the development of HPV-induced OSCC. HIF-1α and HPV16 E7 labeling indexes suggest also a positive association between the two proteins in infected positive HPV16 DNA OSCC. Finally, it may also be extremely interesting to investigate an effective way to inhibit tumor progression in early infected positive HPV16 DNA OSCCs by blocking the activity of HIF-1.

## Methods

### Patients and Tumor Specimens

The study population was composed of 62 randomly selected patients who underwent surgical resection for histologically diagnosed OSCC; tissue samples taken from the subjects and stored in the archives of the Department of Sciences for Health Promotion, Section of Anatomic Pathology, University of Palermo, provided adequate histological material. Twenty-three of the 62 OSCC were positive for HPV DNA; all 23 HPV DNA positive tumors contained HPV16 DNA. Forty-seven cases, 39 negative for HPV DNA and 8 positive for HPV16 DNA, were selected from our previous work [53]; others HPV DNA positive samples were excluded from the study because specific monoclonal antibodies to HPV E7 protein for immunohistochemistry was available only for HPV16 type. The 8 HPV16 positive cases from our previous study were retest to assert their HPV16 positivity via PCR method from paraffin embedded archived material. The remaining 15 cases positive for HPV16 DNA were added from PCR analysis on other archival material.

The mean age of the 62 patients was 62.14 years (SD ± 9.42), range (29-81); males 20 (35.7%), females 36 (64.3%). Out of 62 OSCCs, 29 were localised on the tongue, 15 on buccal mucosa, 11 on alveolar ridge mucosa and 7 on the palate.

Informed consent was obtained from all patients and the IRB of the University of Palermo, Italy, approved the study.

### Histological analysis

Microscopic evaluation was performed by one oral pathologist (VR), who confirmed OSCC diagnosis and also determined the score of Histological Grading (HG). The World Health Organization criteria [54] were used as follows: grade 1 (G1), well differentiated; grade 2 (G2), moderately differentiated and grade 3 (G3), poorly differentiated. Grade is dependent on the degree of prickle formation, keratinization, and overall resemblance of carcinoma to normal squamous epithelium.

### PCR and sequencing analysis

Each formalin-fixed, paraffin-embedded tissue sample was processed as described in[55] to obtain DNA samples to be used in the following PCR analysis. All samples were checked for DNA quality by amplification of the human β-globin gene using the couple of primers PCO3+ (5'CTTCTGACACAACTGTGTTCACTAGC3') and PCO4+ (5'TCACCGCAACTTCATCCACGTTCACC3') and tested in duplicate. Three types of controls were included in each reaction series: blank control, HPV-negative cells Wi as negative control and HPV18 DNA-positive HeLa cells, in dilutions from 20,000-50,000 down to 2-5 HPV-DNA copies, as positive control. HPV-DNA was amplified in a *n*PCR assay [MY09 (CGTCC(AC)A(AG)(AG)GGA(AT)ACTGATC) - MY11 (GC(AC)CAGGG(AT)CATAA(CT)AATGG) degenerated primer pair in combination with GP5 (5'TTTGTTACTGTGGTAGATACTAC3') - GP6 (5'GAAAAATAAACTGTAAATCATATTC3') primer pair] as previously described [56], and amplifications were performed in a DNA thermal cycler (Mastercycler gradient, Eppendorf, Hamburg, Germany); amplification products were analyzed in 1% agarose gel.

HPV genotyping was based on direct sequencing of MY- or MY/GP-PCR fragments [56]. Amplification products were purified by Microcon YM-100 (Amicon, Millipore); the sequence of both DNA strands was determined by the BigDye Ready Reaction Kit (Perkin-Elmer Applied Biosystem, Foster City, Calif.) in the automatic sequencer ABI Prism 310 Analyzer (Perkin-Elmer Applied Biosystem). Alignments were obtained from the GenBank on-line BLAST server and HPV sequences were downloaded from the HPV database http://hpv-web.lanl.gov.

### Immunohistochemistry

Tissue samples were fixed in 10% buffered formalin, dehydrated in ethanol and paraffin-embedded according to the routine technique. Immunohistochemical analyses were performed on 3-μm-thick paraffin-embedded sections of tumors as routine procedure as described in [57]. Briefly, sections were subsequently exposed to either mouse monoclonal anti HPV16 E7 oncoprotein (Invitrogen Corporation, Camarillo, CA, USA), dilution 1:50, for 2 hours at 25°C or rabbit polyclonal anti-HIF-1α (Novus Biologicals, Littleton, Co, USA), dilution 1:100, for 1 hour at 25°C, or unconjugated rabbit immunoglobulins (negative control) for 1 hour at 25°C. Staining was detected using Novolink Polymer Detection System, (Novocastra Laboratories, Newcastle Upon Tyne, UK) according to manufacturer's instructions and counterstained with aqueous hematoxylin.

### Immunohistochemical evaluation

For the evaluation of HPV16 E7 oncoprotein and HIF-1α immunoreactions, tissues were examined for evidence of staining with the aid of the NIH ImageJ software http://rsbweb.nih.gov/ij. Specimens were observed under a light microscope (DM4000B with a plan 40× objective, aperture 0.65, 10× ocular; Leica Microsystems, Wetzlar, Germany), and images of representative fields were captured with a digital camera (DFC480; Leica Microsystems) onto a hard drive disk and then opened with the ImageJ software to evaluate the indices of positive staining that was regarded as Labeling Index (LI). With the aim to use the cell counter technique, positive and negative stained cells were marked placing different colors marks by mouse clicking directly from the screen. For each case a minimum of 10^3 ^cells was counted; ImageJ software was then able to automatically generate the percentage of tumor cells stained with HPV16 E7 and HIF-1α antibodies. Only epithelial cells were counted regardless of localization or intensity of staining. Twenty random cases were evaluated separately by two different observers (V.R. and F.A.); since the variation was less than 5%, the first pathologist's data were used.

### Statistical methods

Continuous variables were analyzed as mean values ± standard deviation (SD). Rates and proportions were calculated for categorical data. For categorical variables, differences were analyzed by the χ^2 ^test and Fisher exact test when appropriate. Normality of distribution for quantitative data was assessed by the Kolmogorov-Smirnov test. Differences between two groups were detected by the unpaired Student's t-test for continuous variables with normal distribution. For continuous variable without normal distribution, we used nonparametric tests, and differences were analyzed by the Mann-Whitney *U *test. Correlations among continuous variables were determined by the use of Spearman test. *p *< .05 was considered statistically significant. All analyses were performed with Statistical Package for Social Science (SPSS for Windows, version 17.0; ©SPSS Inc., Chicago, Ill, USA).

## List of abbreviations

HR: High Risk; HPV: Human PapillomaVirus; OSCC: Oral Squamous Cell Carcinoma; HIF-1α: Hypoxia Inducible Factor-1 alpha; HDAC: Histone DeACetylases; HIF-1β: Hypoxia Inducible Factor-1 beta; VHL: Von Hippel-Lindau; CBP: CREB Binding Protein; SCC: Squamous Cell Carcinoma; PCNA: Proliferating Cell Nuclear Antigen; DFO: DeFeroxamine Mesylate; PCR: Polymerase Chain Reaction; IRB: Institutional Review Board; HG: Histological Grading; nPCR: Nested Polymerase Chain Reaction; LI: Labeling Index; NIH: National Institutes of Health; SD: standard deviation; SPSS: Statistical Package for Social Science.

## Competing interests

The authors declare that they have no competing interests.

## Authors' contributions

VR and GC: were responsible for developing the concept, full proposal development and obtaining ethical approvals. ODF: carried out the field work and was responsible for data collection. WA and FA: made all laboratory analyses and contributed to the interpretation of laboratory results. VR: analyzed the labeling indexes. MCA: provided statistical analyses and interpretation of the results. WA: wrote the manuscript while VR GC FC and GP: gave their critical comments upon the writing process and revised the final manuscript. All authors read and approved the version to be published.

## References

[B1] Smith EM Ritchie JM Summersgill KF Hoffman HT Wang DH Haugen TH Turek LP Human papillomavirus in oral exfoliated cells and risk of head and neck cancer J Natl Cancer Inst 2004 96 449 455 10.1093/jnci/djh074 15026470

[B2] Gillison ML Koch WM Capone RB Spafford M Westra WH Wu L Zahurak ML Daniel RW Viglione M Symer DE Evidence for a causal association between human papillomavirus and a subset of head and neck cancers J Natl Cancer Inst 2000 92 709 720 10.1093/jnci/92.9.709 10793107

[B3] Gillison ML Koch WM Shah KV Human papillomavirus in head and neck squamous cell carcinoma: are some head and neck cancers a sexually transmitted disease? Curr Opin Oncol 1999 11 191 199 10.1097/00001622-199905000-00010 10328594

[B4] Gillison ML Shah KV Human papillomavirus-associated head and neck squamous cell carcinoma: mounting evidence for an etiologic role for human papillomavirus in a subset of head and neck cancers Curr Opin Oncol 2001 13 183 188 10.1097/00001622-200105000-00009 11307062

[B5] Mork J Lie AK Glattre E Hallmans G Jellum E Koskela P Moller B Pukkala E Schiller JT Youngman L Human papillomavirus infection as a risk factor for squamous-cell carcinoma of the head and neck N Engl J Med 2001 344 1125 1131 10.1056/NEJM200104123441503 11297703

[B6] Smith EM Hoffman HT Summersgill KS Kirchner HL Turek LP Haugen TH Human papillomavirus and risk of oral cancer Laryngoscope 1998 108 1098 1103 10.1097/00005537-199807000-00027 9665264

[B7] Smith EM Ritchie JM Summersgill KF Klussmann JP Lee JH Wang D Haugen TH Turek LP Age, sexual behavior and human papillomavirus infection in oral cavity and oropharyngeal cancers Int J Cancer 2004 108 766 772 10.1002/ijc.11633 14696105

[B8] Lindel K Beer KT Laissue J Greiner RH Aebersold DM Human papillomavirus positive squamous cell carcinoma of the oropharynx: a radiosensitive subgroup of head and neck carcinoma Cancer 2001 92 805 813 10.1002/1097-0142(20010815)92:4<805::AID-CNCR1386>3.0.CO;2-9 11550151

[B9] Schwartz SM Daling JR Doody DR Wipf GC Carter JJ Madeleine MM Mao EJ Fitzgibbons ED Huang S Beckmann AM Oral cancer risk in relation to sexual history and evidence of human papillomavirus infection J Natl Cancer Inst 1998 90 1626 1636 10.1093/jnci/90.21.1626 9811312

[B10] Schwartz SR Yueh B McDougall JK Daling JR Schwartz SM Human papillomavirus infection and survival in oral squamous cell cancer: a population-based study Otolaryngol Head Neck Surg 2001 125 1 9 10.1067/mhn.2001.116979 11458206

[B11] Sisk EA Bradford CR Jacob A Yian CH Staton KM Tang G Harris MO Carey TE Lancaster WD Gregoire L Human papillomavirus infection in "young" versus "old" patients with squamous cell carcinoma of the head and neck Head Neck 2000 22 649 657 10.1002/1097-0347(200010)22:7<649::AID-HED2>3.0.CO;2-B 11002318

[B12] Castellsague X Bosch FX Munoz N Environmental co-factors in HPV carcinogenesis Virus Res 2002 89 191 199 10.1016/S0168-1702(02)00188-0 12445659

[B13] Chang JY Lin MC Chiang CP High-risk human papillomaviruses may have an important role in non-oral habits-associated oral squamous cell carcinomas in Taiwan Am J Clin Pathol 2003 120 909 916 10.1309/C5P6NUQ2NW6LCTBP 14671980

[B14] Dahlstrom KR Adler-Storthz K Etzel CJ Liu Z Dillon L El-Naggar AK Spitz MR Schiller JT Wei Q Sturgis EM Human papillomavirus type 16 infection and squamous cell carcinoma of the head and neck in never-smokers: a matched pair analysis Clin Cancer Res 2003 9 2620 2626 12855639

[B15] de Villiers EM Gunst K Stein H Scherubl H Esophageal squamous cell cancer in patients with head and neck cancer: Prevalence of human papillomavirus DNA sequences Int J Cancer 2004 109 253 258 10.1002/ijc.11685 14750177

[B16] Herrero R Castellsague X Pawlita M Lissowska J Kee F Balaram P Rajkumar T Sridhar H Rose B Pintos J Human papillomavirus and oral cancer: the International Agency for Research on Cancer multicenter study J Natl Cancer Inst 2003 95 1772 1783 14652239 10.1093/jnci/djg107

[B17] Clifford GM Smith JS Plummer M Munoz N Franceschi S Human papillomavirus types in invasive cervical cancer worldwide: a meta-analysis Br J Cancer 2003 88 63 73 10.1038/sj.bjc.6600688 12556961 PMC2376782

[B18] Walboomers JM Jacobs MV Manos MM Bosch FX Kummer JA Shah KV Snijders PJ Peto J Meijer CJ Munoz N Human papillomavirus is a necessary cause of invasive cervical cancer worldwide J Pathol 1999 189 12 19 10.1002/(SICI)1096-9896(199909)189:1<12::AID-PATH431>3.0.CO;2-F 10451482

[B19] Kreimer AR Clifford GM Boyle P Franceschi S Human papillomavirus types in head and neck squamous cell carcinomas worldwide: a systematic review Cancer Epidemiol Biomarkers Prev 2005 14 467 475 10.1158/1055-9965.EPI-04-0551 15734974

[B20] Termine N Panzarella V Falaschini S Russo A Matranga D Lo Muzio L Campisi G HPV in oral squamous cell carcinoma vs head and neck squamous cell carcinoma biopsies: a meta-analysis (1988-2007) Ann Oncol 2008 19 1681 1690 10.1093/annonc/mdn372 18558666

[B21] Azzimonti B Pagano M Mondini M De Andrea M Valente G Monga G Tommasino M Aluffi P Landolfo S Gariglio M Altered patterns of the interferon-inducible gene IFI16 expression in head and neck squamous cell carcinoma: immunohistochemical study including correlation with retinoblastoma protein, human papillomavirus infection and proliferation index Histopathology 2004 45 560 572 10.1111/j.1365-2559.2004.02000.x 15569046

[B22] Dai M Clifford GM le Calvez F Castellsague X Snijders PJ Pawlita M Herrero R Hainaut P Franceschi S Human papillomavirus type 16 and TP53 mutation in oral cancer: matched analysis of the IARC multicenter study Cancer Res 2004 64 468 471 10.1158/0008-5472.CAN-03-3284 14744758

[B23] Munger K Baldwin A Edwards KM Hayakawa H Nguyen CL Owens M Grace M Huh K Mechanisms of human papillomavirus-induced oncogenesis J Virol 2004 78 11451 11460 10.1128/JVI.78.21.11451-11460.2004 15479788 PMC523272

[B24] Bodily JM Mehta KP Laimins LA Human papillomavirus E7 enhances hypoxia-inducible factor 1-mediated transcription by inhibiting binding of histone deacetylases Cancer Research 2011 71 1187 1195 10.1158/0008-5472.CAN-10-2626 21148070 PMC3077548

[B25] Hanahan D Folkman J Patterns and emerging mechanisms of the angiogenic switch during tumorigenesis Cell 1996 86 353 364 10.1016/S0092-8674(00)80108-7 8756718

[B26] Brat DJ Kaur B Van Meir EG Genetic modulation of hypoxia induced gene expression and angiogenesis: relevance to brain tumors Front Biosci 2003 8 d100 116 10.2741/942 12456339

[B27] Bardos JI Ashcroft M Negative and positive regulation of HIF-1: a complex network Biochim Biophys Acta 2005 1755 107 120 15994012 10.1016/j.bbcan.2005.05.001

[B28] Ruas JL Poellinger L Hypoxia-dependent activation of HIF into a transcriptional regulator Semin Cell Dev Biol 2005 16 514 522 10.1016/j.semcdb.2005.04.001 15908239

[B29] Schmid T Zhou J Brune B HIF-1 and p53: communication of transcription factors under hypoxia J Cell Mol Med 2004 8 423 431 10.1111/j.1582-4934.2004.tb00467.x 15601571 PMC6740063

[B30] Ruas JL Berchner-Pfannschmidt U Malik S Gradin K Fandrey J Roeder RG Pereira T Poellinger L Complex regulation of the transactivation function of hypoxia-inducible factor-1 alpha by direct interaction with two distinct domains of the CREB-binding protein/p300 J Biol Chem 2010 285 2601 2609 10.1074/jbc.M109.021824 19880525 PMC2807317

[B31] Chang CC Lin BR Chen ST Hsieh TH Li YJ Kuo MY HDAC2 promotes cell migration/invasion abilities through HIF-1alpha stabilization in human oral squamous cell carcinoma J Oral Pathol Med 2011 40 567 575 10.1111/j.1600-0714.2011.01009.x 21332579

[B32] Bodily JM Mehta KP Laimins LA Human papillomavirus E7 enhances hypoxia-inducible factor 1-mediated transcription by inhibiting binding of histone deacetylases Cancer Res 2011 71 1187 1195 10.1158/0008-5472.CAN-10-2626 21148070 PMC3077548

[B33] Fiedler M Muller-Holzner E Viertler HP Widschwendter A Laich A Pfister G Spoden GA Jansen-Durr P Zwerschke W High level HPV-16 E7 oncoprotein expression correlates with reduced pRb-levels in cervical biopsies Faseb J 2004 18 1120 1122 15155561 10.1096/fj.03-1332fje

[B34] Ha PK Califano JA The role of human papillomavirus in oral carcinogenesis Crit Rev Oral Biol Med 2004 15 188 196 10.1177/154411130401500402 15284184

[B35] Fakhry C Westra WH Li S Cmelak A Ridge JA Pinto H Forastiere A Gillison ML Improved survival of patients with human papillomavirus-positive head and neck squamous cell carcinoma in a prospective clinical trial J Natl Cancer Inst 2008 100 261 269 10.1093/jnci/djn011 18270337

[B36] Li W Thompson CH O'Brien CJ McNeil EB Scolyer RA Cossart YE Veness MJ Walker DM Morgan GJ Rose BR Human papillomavirus positivity predicts favourable outcome for squamous carcinoma of the tonsil Int J Cancer 2003 106 553 558 10.1002/ijc.11261 12845651

[B37] Mellin H Friesland S Lewensohn R Dalianis T Munck-Wikland E Human papillomavirus (HPV) DNA in tonsillar cancer: clinical correlates, risk of relapse, and survival Int J Cancer 2000 89 300 304 10.1002/1097-0215(20000520)89:3<300::AID-IJC14>3.0.CO;2-G 10861508

[B38] Soares CP Benatti Neto C Fregonezi PA Teresa DB Santos RT Longatto Filho A Maeda MY Computer-assisted analysis of p53 and PCNA expression in oral lesions infected with human papillomavirus Anal Quant Cytol Histol 2003 25 19 24 12630078

[B39] Richardson H Kelsall G Tellier P Voyer H Abrahamowicz M Ferenczy A Coutlee F Franco EL The natural history of type-specific human papillomavirus infections in female university students Cancer Epidemiol Biomarkers Prev 2003 12 485 490 12814991

[B40] Stanley M Immunobiology of HPV and HPV vaccines Gynecol Oncol 2008 109 S15 21 10.1016/j.ygyno.2008.02.003 18474288

[B41] Park RB Androphy EJ Genetic analysis of high-risk e6 in episomal maintenance of human papillomavirus genomes in primary human keratinocytes J Virol 2002 76 11359 11364 10.1128/JVI.76.22.11359-11364.2002 12388696 PMC136782

[B42] Thomas M Pim D Banks L The role of the E6-p53 interaction in the molecular pathogenesis of HPV Oncogene 1999 18 7690 7700 10.1038/sj.onc.1202953 10618709

[B43] Kadaja M Isok-Paas H Laos T Ustav E Ustav M Mechanism of genomic instability in cells infected with the high-risk human papillomaviruses PLoS Pathogens 2009 5 e1000397 10.1371/journal.ppat.1000397 19390600 PMC2666264

[B44] zur Hausen H Papillomaviruses and cancer: from basic studies to clinical application Nature Reviews Cancer 2002 2 342 350 10.1038/nrc798 12044010

[B45] Hanahan D Weinberg RA The hallmarks of cancer Cell 2000 100 57 70 10.1016/S0092-8674(00)81683-9 10647931

[B46] Smith-McCune K Zhu YH Hanahan D Arbeit J Cross-species comparison of angiogenesis during the premalignant stages of squamous carcinogenesis in the human cervix and K14-HPV16 transgenic mice Cancer Res 1997 57 1294 1300 9102216

[B47] Smith-McCune KK Weidner N Demonstration and characterization of the angiogenic properties of cervical dysplasia Cancer Res 1994 54 800 804 7508337

[B48] Tang X Zhang Q Nishitani J Brown J Shi S Le AD Overexpression of human papillomavirus type 16 oncoproteins enhances hypoxia-inducible factor 1 alpha protein accumulation and vascular endothelial growth factor expression in human cervical carcinoma cells Clin Cancer Res 2007 13 2568 2576 10.1158/1078-0432.CCR-06-2704 17473185

[B49] Clere N Bermont L Fauconnet S Lascombe I Saunier M Vettoretti L Plissonnier ML Mougin C The human papillomavirus type 18 E6 oncoprotein induces Vascular Endothelial Growth Factor 121 (VEGF121) transcription from the promoter through a p53-independent mechanism Exp Cell Res 2007 313 3239 3250 10.1016/j.yexcr.2007.06.029 17678892

[B50] Chen W Li F Mead L White H Walker J Ingram DA Roman A Human papillomavirus causes an angiogenic switch in keratinocytes which is sufficient to alter endothelial cell behavior Virology 2007 367 168 174 10.1016/j.virol.2007.05.030 17602722 PMC2043482

[B51] Toussaint-Smith E Donner DB Roman A Expression of human papillomavirus type 16 E6 and E7 oncoproteins in primary foreskin keratinocytes is sufficient to alter the expression of angiogenic factors Oncogene 2004 23 2988 2995 10.1038/sj.onc.1207442 14968115

[B52] Nakamura M Bodily JM Beglin M Kyo S Inoue M Laimins LA Hypoxia-specific stabilization of HIF-1alpha by human papillomaviruses Virology 2009 387 442 448 10.1016/j.virol.2009.02.036 19321184 PMC2674135

[B53] Campisi G Giovannelli L Calvino F Matranga D Colella G Di Liberto C Capra G Leao JC Lo Muzio L Capogreco M D'Angelo M HPV infection in relation to OSCC histological grading and TNM stage. Evaluation by traditional statistics and fuzzy logic model Oral Oncol 2006 42 638 645 10.1016/j.oraloncology.2005.11.007 16483833

[B54] Barnes LEJ Reichart P Sidransky D Head and Neck Tumours. Pathology and Genetics. WHO Classification of Tumours 2005 Lyon: IARC Press

[B55] Pikor LA Enfield KS Cameron H Lam WL DNA extraction from paraffin embedded material for genetic and epigenetic analyses J Vis Exp 2011, (49) 21490570 10.3791/2763 PMC3197328

[B56] Giovannelli L Campisi G Lama A Giambalvo O Osborn J Margiotta V Ammatuna P Human papillomavirus DNA in oral mucosal lesions J Infect Dis 2002 185 833 836 10.1086/339193 11920302

[B57] Zerilli M Zito G Martorana A Pitrone M Cabibi D Cappello F Giordano C Rodolico V BRAF(V600E) mutation influences hypoxia-inducible factor-1alpha expression levels in papillary thyroid cancer Mod Pathol 2010 23 1052 1060 10.1038/modpathol.2010.86 20473281

